# Sarcina ventriculi a rare pathogen

**DOI:** 10.4322/acr.2021.337

**Published:** 2021-10-18

**Authors:** Luciano Paludo Marcelino, Dirceu Felipe Valentini, Simone Márcia dos Santos Machado, Pedro Guilherme Schaefer, Raquel Camara Rivero, Alessandro Bersch Osvaldt

**Affiliations:** 1 Universidade Federal do Rio Grande do Sul (UFRGS), Medical School, Porto Alegre, RS, Brasil.; 2 Universidade Federal do Rio Grande do Sul (UFRGS), Hospital de Clínicas de Porto Alegre, Service of Digestive Surgery, Porto Alegre, RS, Brasil; 3 Universidade Federal do Rio Grande do Sul (UFRGS), Hospital de Clínicas de Porto Alegre, Service of Pathology, Porto Alegre, RS, Brasil; 4 Universidade Federal do Rio Grande do Sul (UFRGS), Hospital de Clínicas de Porto Alegre, Service of Digestive Surgery, Group for Biliary Tract and Pancreas, Porto Alegre, RS, Brasil.

**Keywords:** Sarcina, Clostridium, Gastroparesis

## Abstract

*Sarcina ventriculi* is a gram-positive bacterium, able to survive in extreme low pH environment. It’s first description dates from 1842, by John Goodsir. Since then, just a few cases have been reported. In veterinary medicine, especially in ruminants, it causes bloating, vomiting, gastric perforation and death of the animal. It is commonly associated with delayed gastric emptying or obstruction to gastric outlet, although it’s pathogenicity in humans is not fully understood. We report two cases with identification of the bacteria in gastric specimens stained with hematoxylin-eosin staining, in different clinical settings. The first patient is a young female patient, presenting cardiac arrest and death after gastric perforation and the second patient an adult male presenting with gastric adenocarcinoma, treated with partial gastrectomy followed by adjuvant chemoradiation. In our literature review, we identified forty-five cases reporting *Sarcina ventriculi* appearance, with a sudden increase since 2010.

## INTRODUCTION

*Sarcina ventriculi* is a gram-positive anaerobic bacterium, with carbohydrate fermentative metabolism, tetrad characteristic morphology and able to survive in extreme low pH environment.^1^^,^ 2 It was first described by the Scottish anatomist and biologist John Goodsir,^3^ in 1842, after the microscopic analysis of the gastric content of a patient with daily vomiting.

In veterinary medicine, it has been associated with bloating, vomiting, emphysematous gastritis and death of the animal, especially goats, calves, lambs and equines.^4^^,^ 5 Despite these well-known conditions in other animals, the pathogenicity in humans is not fully understood, and it has been identified associated with varied clinical features; from asymptomatic patients^6^ to patients with gastric perforation.^7^

There are few case reports with this bacterium identification. Here, we report two cases with *Sarcina ventriculi* identification with distinct clinical settings. Two independent authors performed a review of the literature, through EMBASE and MEDLINE databases, using the following MeSH Terms: *Sarcina ventriculi* and *Clostridium ventriculi*. Additionally, the reference lists of the selected articles were individually examined to obtain other potentially relevant studies. Only articles in English were included. We identified thirty-seven articles, reporting forty-five cases until July 2020, with an increasing description since 2010 (Table 1). In most cases, the clinical conditions resulted in slower gastric emptying, such as previous surgery, obstruction or gastroparesis.

**Table 1:** List of cases reported with *Sarcina ventriculi* identification since January 2003 until July 2020

**Ref.**	**Age (y) Sex**	**Site**	**Clinical Features**
**^7^**	14/M	S	Gastric perforation. Recovery after laparotomy and gentamicin and metronidazole.
	50/M	D	Duodenal mass, biopsy showed chronic superficial gastritis and *Sv*.
**^8^**	3/F	S	Abdominal distention. EGD with fluid, air and partial gastric necrosis. *Sv* in gastric biopsy, and *Candida sp.* in the stomach and esophagus. Recovery after imipenem, fluconazole and omeprazole.
**^9^**	58/F	S	EGD - biopsy revealing bezoar and pyloric mass - active chronic gastritis and *Sv*. Endoscopy after one month - complete obstruction caused by a pyloric mass - adenocarcinoma
	44/F	S	Gastroparesis. EGD - biopsy with gastric ulcer with *Sv* and pyloric hyperplastic polyps. Improvement of symptoms after treatment with omeprazole, ranitidine and metoclopramide.
	36/M	S	Gastroparesis related to narcotics, retained food and *SV* after EGD. Repeated biopsy was negative for *Sv* after six weeks with feeding through jejunostomy tube.
	12/F	S	Esophageal atresia and gastric pull through with anastomotic narrowing. Presented with dysphagia and retained food at EGD. Biopsy revealing reflux esophagitis and *Sarcina ventriculi*.
	46/F	S	Pylorus-preserving pancreaticoduodenectomy (pancreatic adenocarcinoma), retained food and *Sv* after EGD with biopsy.
**^10^**	73/M	S	Previous antrectomy with vagotomy and Billroth II reconstruction. EGD performed in investigation of anemia, showed retained food, polyps and diffuse gastric erythema. Gastric biopsy with inflammation, ulcer, bacterial overgrowth *So*. Treatment with metronidazole and ciprofloxacin.
**^11^**	12/M	E, S, D	Emesis and epigastric pain, EGD with erosive esophagitis, erythematous gastric mucosa and edematous pylorus. *So* in association with active erosive esophagitis, chronic active *H.pylori* gastritis and *H. pylori* duodenitis.
	16/F	E, S	GERD. EGD with food debris, erosive esophagitis and edematous pylorus. *So* in and erosive esophagitis, chronic active *H. pylori* gastritis and *H. pylori* duodenitis.
**^12^**	48/F	B	Congenital Chloride Diarrhea and episode of vomiting and fever. Anaerobic blood culture revealed gram-positive cocci, and sequence of the 16S rRNA compatible with *Sv* was identified. Recovery after treatment with amoxicillin.
**^13^**	34/F	S	Epigastric pain, EGD showed normal mucosa with *So*. Treated with ciprofloxacin and metronidazole.
**^14^**	50/M	S	Chronic alcoholic and virus C hepatitis, jaundice, fever and abdominal pain. EGD with food residue and mucosal edema. Biopsy revealed intestinal metaplasia and presence of *Sv.* Recovery after treatment with ciprofloxacin, metronidazole and sucralfate.
**^15^**	3/M	D	Diarrhea, one month after treatment for acute viral hepatitis. EGD with mild grooving in duodenum, and biopsy with *So* and *Giardia* microorganisms
**^16^**	16/M	S	Diarrhea, abdominal pain and nausea. EGD with food residues, esophagitis, pangastritis and scalloping of duodenal folds. Biopsy confirmed celiac disease. Gastric biopsy with lymphoplasmacytic cells and *So*.
**^17^**	37/F	S	Cystic Fibrosis, intermittent epigastric pain and delayed gastric emptying. EGD with erythema of the antrum. Biopsy with moderate chronic gastritis, *So* and *Candida* species.
**^18^**	70/M	E	c-ANCA positive vasculitis, type II diabetes mellitus and chronic obstructive pulmonary disease. Anemia after administration of prednisone and rituximab. EGD with white plaques and esophageal pneumatosis. Biopsy showed superficial acute inflammation and *So*.
**^6^**	57/F	S	Type II diabetes mellitus and hypothyroidism. *Helicobacter pylori* and gastritis treatment. EGDs revealed scarred pylorus, and a pre-pyloric ulcer, with *So on* microscopic examination.
**^19^**	55/F	S	Vomiting and abdominal pain, EGD revealed pyloric ulcer. Fine-needle aspiration and biopsies showed features of gastric adenocarcinoma and *So*.
**^20^**	65/F	S	Previous bariatric surgery. Anemia, EGD and colonoscopy with gastric ulcerations, arteriovenous malformations, diverticulosis and internal hemorrhoids. *Sv* in the gastric biopsy.
**^21^**	43/M	L	Type II diabetes mellitus, pulmonary gangrene, left pneumonectomy. Microscopic examination revealed the presence of polymicrobial infection with aerobes, anaerobes (including *So)* and fungus.
**^22^**	32/F	S	Previous bariatric procedure, anemia and dark stools. EGD with cardia ulcer. Biopsy with *So*. Treated with fluorquinolone, metronidazole and PPI. Emergent laparotomy was performed after perforation during a follow-up EGD.
**^23^**	53/F	S	Previous bariatric procedure with subsequent pouch ulcer, epigastric pain and vomiting. EGD with retained food, polypoid mucosal and a healed ulcer. Biopsy showed chronic gastritis and *So*. Treated with metronidazole.
**^24^**	78/M	S, D	Hiatoplasty and palliative chemo-radiotherapy for gastroesophageal junction adenocarcinoma. EGD with esophageal dilatation and retained food. Biopsy with *So* gastric and duodenum, and recurrent adenocarcinoma at esophageal biopsy.
**^25^**	1/M	U	Stricture of membranous urethra, five months after transurethral fulguration for posterior urethral membrane and vesicoureteric reflux. Urine aspirated through suprapubic aseptic aspiration revealed *So*, treated with ciprofloxacin and metronidazole.
**^26^**	43/F	S	Bariatric procedure, with abdominal pain and tachycardia. CT with massive stomach dilatation, and gastric pneumatosis. Gastrectomy. Microscopic examination showed ischemic injury, transmural gastric necrosis and *Sv.*
**^27^**	65/F	E, S	Metastatic breast cancer and Schatzki ring history. Dysphagia. EGD with esophageal stenosis, 7-mm nodule at the gastroesophageal junction, gastric retained food and gastric ulcers. Biopsy with acute and chronic inflammation, and *Sv*. Treated with esophageal dilatation and stent, PPI, ciprofloxacin and metronidazole
**^28^**	12/F	E, S	Psychomotor retardation, epilepsy, PEG, previous treatments for *H. pylori* gastritis. Dehydration, vomiting and hematemesis. EGD with erosive esophagitis, hemorrhagic gastritis, antral ulcers and retained food. Biopsy with ulcerative esophagitis and gastritis and *Sv.* Treated with ciprofloxacin and metronidazole.
	15/F	S	Neurological impairment, epilepsy and PEG. Respiratory failure with aspiration pneumonia. EGD with mid-esophageal stenosis. Endoscopy by the gastric fistula with erosive gastritis, gastroesophageal junction ulcer and gastric content. Gastric biopsies with gastritis and *SV.* Treated with omeprazole, ciprofloxacin and metronidazole.
**^29^**	10/M	D	Weakness, abdominal distention, chronic diarrhea, anemia, hypoalbuminemia and low serum IgA. EGD with thinning and scalloping of duodenal folds. Biopsy with Celiac Disease, and *Sv*, treated with antibiotic and gluten-free diet.
**^30^**	87/M	S	Dementia and dual antiplatelet therapy to CAD. Abdominal pain and vomiting. CT with gastric emphysema and portal-mesenteric venous gas. Laparotomy negative for bowel ischemia. EGD with erosive and necrotic gastritis. Gastric biopsy with phlegmonous gastritis and *SV*. Treated with fluids, PPI and antibiotics.
**^31^**	48/F	E	Type -2 DM with diabetic enteropathy, presented with abdominal pain, nausea and vomiting. EGD showed esophagitis, and esophageal brushing cytology with *Sv*.
**^32^**	45/F	S	Abdominal discomfort, vomiting, weight loss. EGD with edematous antrum. Gastric brushing cytology with epithelial cells with large nuclei. Biopsy revealed with gastritis, and *Sarcina ventriculi* and *Candida*. Treated with ciprofloxacin, metronidazole and PPI.
**^33^**	59/M	S	Nausea and epigastric pain. CT with gastric pneumatosis and portal venous air. EGD with gastritis and ulcer. Biopsy with chronic gastritis with *So* and *Candida* spp. Treated with Ciprofloxacin, metronidazole and PPI. CT scan was normal after treatment.
**^34^**	38/M	S, D	Hodgkin’s disease in remission. Nausea, emesis, hematemesis and early satiety. EGD with pre-pyloric ulcer and another ulcer extending into the duodenum. Biopsy with gastric with signet ring adenocarcinoma. A brushing of the pyloric/duodenal ulcer showed *Sc*.
**^35^**	86/F	S	Type-2 DM with abdominal pain, nausea and black diarrhea. CT scan revealed gastric pneumatosis and portal vein gas. Hematemesis and hemodynamically instability and death. A postmortem review with emphysematous gastritis with bacterial overgrowth by *Sv.*
**^36^**	70/M	S	Esophagitis and fundoplication. EGD with ulcers and gastric food retention, Barrett’s esophagus. Biopsies with *Sv.* Treated with metronidazole and ciprofloxacin without relief neither bacterium eradication. Surgical revision with improvement.
**^37^**	65/M	B	Bone fractures. diabetes mellitus, heart failure, atrial flutter, pacemaker. Abdominal pain and distention. CT with colonic distention without obstruction with risk of cecal rupture. Laparotomy - ileocecal resection. Two pairs or blood cultures isolated *Sv.*
**^38^**	14/F	S	Esophageal atresia with gastric pull up repair. Recurrent distal esophageal strictures due to GERD. The stricture recurred 6 months after fundoplication. Biopsies with *S*o. Treated with ciprofloxacin and metronidazole, the gastroscopy with resolution of inflammation and *So*.
**^39^**	69/M	S	Type-2 diabetes, small intestinal bacterial overgrowth and pancreatic insufficiency, biliary pancreatitis. Weight loss. EGD with esophagitis, tight pylorus obstruction with hypertrophic inflammatory tissue and a large amount of retained food. *Sv* was by FNA of the pancreas and gastric biopsies. Treated with ciprofloxacin and metronidazole with eradication of the bacteria.
**^40^**	76/M	S	Abdominal pain, rebound tenderness, altered mental status, metabolic acidosis. CT with pneumoperitoneum. Laparotomy - gastric dilatation with rupture. The patient died. Gastric biopsies with *Sv*.
**^41^**	67,/F	E, S	Hiatal hernia with intrathoracic gastric fundus. Recurrent symptoms of GERD. EGD with erosions at the gastroesophageal junction. Biopsies with ulcerative esophagitis and *Sv*. Treated with PPI and domperidone with relief of symptoms. *Sv* remained positive. Control study with atonic stomach with delayed gastric emptying.
**^42^**	13/F	S	Phenylketonuria with PEG feeding tube at five years of age. The histological examination ant the time of the fistula closure, revealed acute and chronic inflammation and colonization with *Sv.*
**^43^**	15/F	S	Rett syndrome profound neurodevelopmental disorder. Abdominal pain and distension. CT with gastric dilation and portal and splenic venous gas. Recovered from cardiac arrest. Laparotomy with dilated stomach with 2cm linear perforation. Gastric specimen examination with *Sv*.

CAD= coronary artery disease; CT=computed tomography), D= duodenum; DM= diabetes mellites; E=esophagus; EGD=esophagogastroduodenoscopy; F= female, FNA= fine needle aspiration; M=male; PEG=percutaneous endoscopic gastrostomy; PPI=proton pump inhibitor, Ref.= reference; S= stomach; *So= Sarcina organism, Sv= Sarcina ventriculi*; y= year. GERD = gastroesophageal reflux disease.

## CASE REPORT

### Case 1

A 15-year-old female patient was brought to the Emergency Room complaining of abdominal pain in the past two days with progressive worsening. At admission she was receiving saline solution through an intraosseous access and oxygen supplementation by a Venturi mask. Blood pressure was 90/60mmHg, heart rate was 130 beats per minute, respiratory rate was 28 movements per minute, afebrile, with clinical signs of poor peripheral perfusion. Diffuse peritoneal irritation signs were identified at abdominal examination.

Fluid resuscitation and antibiotic administration were promptly administered. Arterial blood gas analysis showed mixed acidosis, hyperkalemia and hyperlactatemia. The surgeon on call indicated the exploratory laparotomy; however, fifteen minutes after the arrival at the emergency department, the patient collapsed with pulseless electrical activity (PEA). Cardiopulmonary resuscitation maneuvers and orotracheal intubation were held. At this moment, an abdominal puncture with an over-the-needle catheter was performed and the drainage of gas and a brownish liquid was observed.

Despite the advanced life support, she died 1 hour after the admission. During the resuscitation maneuvers, a laparotomy was performed at bedside with identification of hemoperitoneum, and no report of pus or enteric secretion.

**Figure 1:**
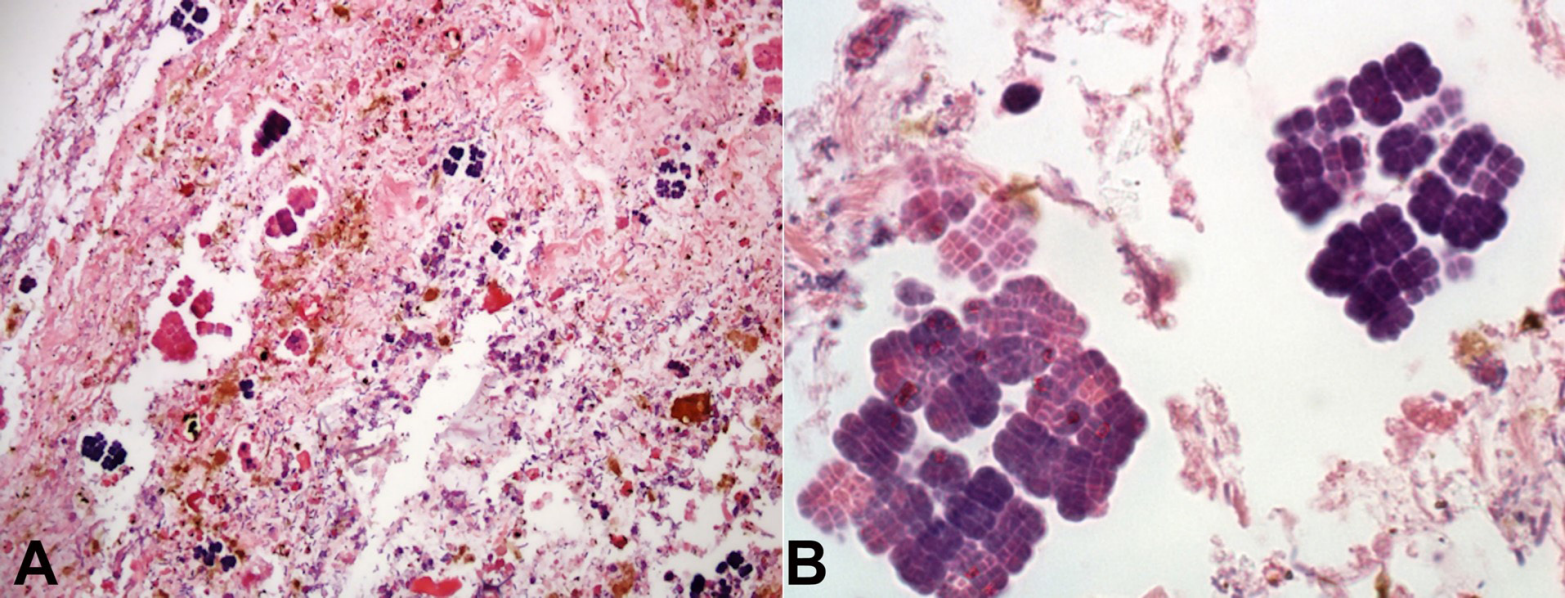
Photomicrograph of the stomach. **A** – *Sarcina ventriculi* identification in stomach specimen sampled during necropsy (H&E, 10x); **B** – *Sarcina ventriculi* identification in stomach specimen sampled during necropsy (H&E, 100x).

Patient’s body was sent to autopsy after the family authorization. Macroscopically there was a 6cm perforation at the transition of the body and antrum of the stomach, and the gastric mucosa was congested and hemorrhagic. Microscopic revealed ischemic necrosis of the stomach wall, polymorphonuclear cells in the *lamina propria* and inside vessels of mucosa and submucosa, and bacterial colonies close to the perforation site, formed by basophilic cells, arranged in cuboids, tetrahedral structures, compatible with the diagnosis of *Sarcina ventriculi* (Figure 1A and 1B).

### Case 2

**Figure 2:**
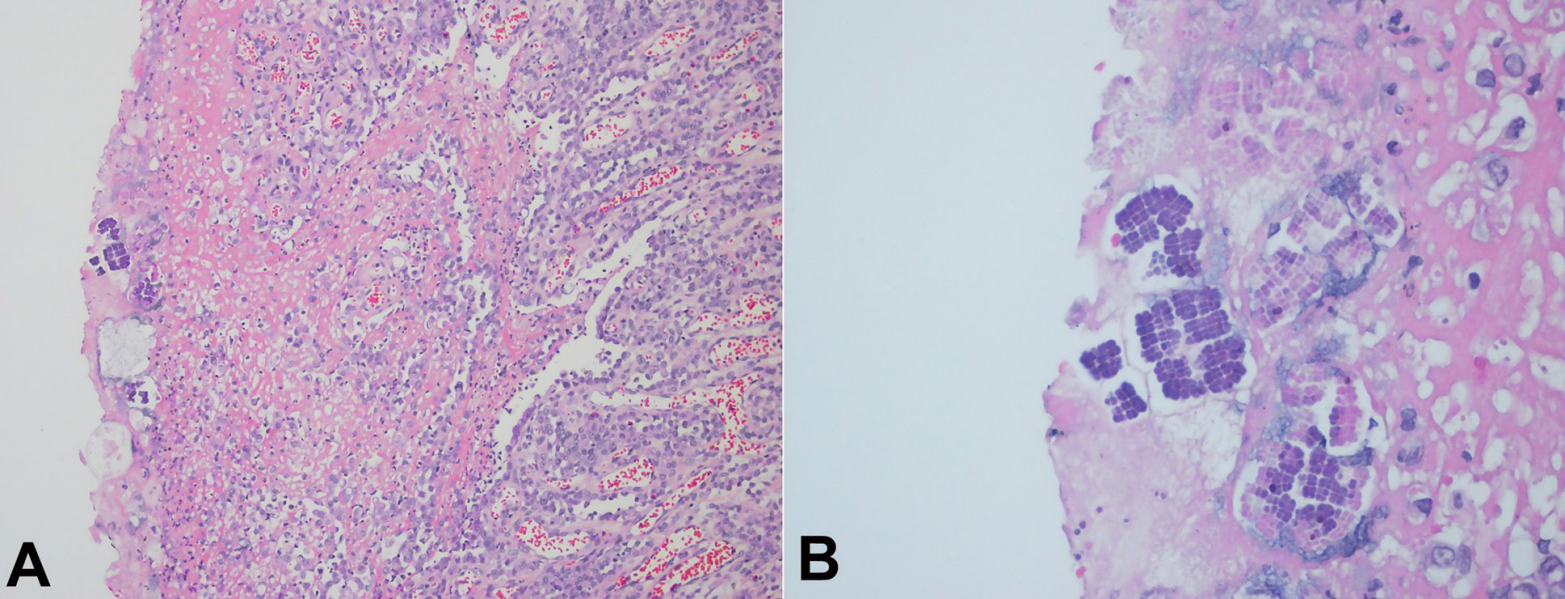
Photomicrograph of the stomach. **A** – *Sarcina ventriculi* identification in stomach specimen after gastrectomy (H&E, 10x); **B** – *Sarcina ventriculi* identification in stomach specimen after gastrectomy (H&E, 40x).

A 58-year-old male patient, a former smoker, was submitted to an upper gastrointestinal endoscopy due to epigastric pain in the past three months. Hemoglobin level was 10.8g/dL (reference range 12.8-17.8g/dL), and no other laboratory abnormality was detected. The upper gastrointestinal endoscopy demonstrated a non-obstructive neoplastic lesion in the antrum of the stomach, confirmed by biopsy as a diffuse gastric adenocarcinoma with signet-ring cells. CT scan revealed no distant metastasis, and patient was submitted to a partial gastrectomy. Anatomopathological analysis of the specimen confirmed a poorly differentiated adenocarcinoma, with 5.5cm at the distal stomach and extending through all gastric walls. There were free resection margins and no metastasis was seen in any of the seventeen lymph nodes (pT3 pN0). *Sarcina ventriculi* was identified at the mucosa (Figure 2A and 2B). The patient had a good recovery after the surgery, but was readmitted due to a wound infection, treated with local drainage and antibiotic therapy. He is currently receiving postoperative chemoradiotherapy (MacDonald regimen).

## DISCUSSION

*Sarcina ventriculi* was first described in 1842 by the Scottish anatomist and microbiologist John Goodsir,^3^ after microscopic analysis of the emetic content of a 19-year-old patient, later described as *sarcinous vomiting*. In 1872, David Ferrier visualized the bacterium in blood samples from patients with enteric fever,^44^ but for a few decades it was discussed whether it was really a living being or not. In 1911, however, it was isolated after culture with anaerobic techniques by Beijerinck,^45^ putting an end to the doubts about its real nature.

*Sarcina ventriculi* is a gram-positive bacterium, able to survive in extreme low pH environment, dependent of carbohydrate fermentation – its sole energy source – with subsequent production of carbon dioxide, ethanol, hydrogen and acetic acid.^1^^,^ 2 Its morphological characteristics are the nearly spherical shape, individual size ranging from 1.8 to 3μm, refractory nature, basophilic staining to hematoxylin-eosin, flattening of walls between cells and presence of extracellular cellulose. It occurs in a characteristic packet-forming, tetrads or packets of eight or more, resulting from division into perpendicular planes.^1^^,^ 46 Although similar to the *Micrococcus* species, some morphological characteristics help distinguish them, such as a larger size, spore formation, and catalase negative reaction. In most cases, optical microscopy with hematoxylin-eosin and Gram staining is enough for the diagnosis, and additional stains such as Brown and Hopps can help to identify to highlight the tetrad morphology.^9^ Polymerase Chain Reaction (PCR) and sequencing of 16S rRNA of the bacterium or pyruvate decarboxylase genes may also be performed, although the molecular confirmation of the diagnosis is not routinely necessary, and the diagnosis is based on the histological findings.^9^^,^ 46

Recently, based on studies with comparisons of 16S rRNA gene sequences of gram-positive anaerobic bacteria, Lawson and Rainey^47^ have proposed that the genus *Sarcina* should be transferred to the genus *Clostridium* cluster I. The genus *Sarcina* had two species, *S. ventriculi* and *S. maxima*, being described now as *Clostridium ventriculi* comb. nov. and *Clostridium maximum* comb. nov., respectively.

The natural habitat of the bacteria is the soil, and is also found in water and air, in the form of spores.^2^ In veterinary medicine, especially among ruminants, it causes gastric dilatation, vomiting and emphysematous gastritis, and may be a cause of death in these animals.^4^ The infection of humans or other animals is caused by the contamination of food. Also, it has been demonstrated the relationship between the consumption of vegetarian diet and the presence of the bacteria in the feces.^48^ The pathogenicity of *Sarcina ventriculi* in humans is not fully understood, and it has been isolated from different sites of the gastrointestinal, urinary and respiratory tracts, as wells as in blood, in different clinical settings.^12^^,^ 21 ^,^ 25 There is a hypothesis that the bacteria could damage the gastrointestinal mucosa through the accumulation of acetaldehyde.^46^

In our literature review, we identified forty-five cases reports, and here we described two more cases of the bacterial diagnosis, identified by microscopic morphology. *Sarcina ventriculi* has been identified in any age range from 1 to 87 years-old but occurs mainly in middle age adults (Table 1). There is a slightly more common occurrence between women with 26 cases (55%) compared to 21 cases in men (45%). Most of these reports are from the United States, but there are also cases in Canada, India, Europe and Australia. The two cases reported here are the first from South America.

The most common symptoms were epigastric pain in 24 patients (51%) and nausea and vomiting in 22 patients (47%). The clinical presentation of these cases is also heterogeneous, from asymptomatic (two cases)^6^^,^ 42 to patients with life-threatening situations as hemodynamic instability secondary to emphysematous gastritis (two cases)^26^^,^ 35 and gastric perforation (four cases).^7^^,^ 40 ^,^ 43 Emergency laparotomy was performed in six cases (13%) in the setting of acute abdomen.

In most cases, the bacterial presence is associated with clinical conditions causing gastroesophageal content stasis (26 cases, 55%), such as gastric outlet mechanical obstruction, gastroparesis or delayed gastric emptying. The stomach is the most frequent site of bacterial identification (36 cases, 77%), followed by esophagus (7 cases, 15%) and duodenum (6 cases, 13%). The bacteria were also identified in the blood (two reports)^12^^,^ 37 and urine (one report)^25^ cultures. There is a report of the bacterium found in a pneumonectomy specimen.^21^ In seven cases, the bacteria were found in two or more sites. During the upper gastrointestinal endoscopic exam, the most common finding is retained food or bezoar, associated or not with mucosal damage or ulcer.

In the medical literature, the cases reported are associated with a variety of anatomical or functional conditions associated with gastric stasis, such as: gastric or esophageal adenocarcinoma in five cases,^9^^,^ 19 ^,^ 24 ^,^ 34 laparoscopic gastric banding in four cases,^20^^,^ 22 ^,^ 23 ^,^ 26 post gastrectomy with gastroenteroanastomosis and vagotomy,^10^ psychomotor retardation or neurological deficit with gastrostomy for feeding in three cases,^28^^,^ 42 benign pyloric or duodenal mass,^7^^,^ 9 ^,^ 11 ^,^ 32 Schatzki's ring,^27^ gastroparesis secondary to diabetes mellitus; 6 ^,^ 9 ^,^ 31 among other conditions.

Although most cases are reported in adult patients, the pediatric population is also affected.^8^^,^ 15 Two cases reporting the identification of *Sarcina ventriculi* in patients with Celiac Disease, and other case identified it in a gastric biopsy of a patient with Cystic Fibrosis, strongly suggesting that gastrointestinal dysmotility and intestinal mucosa with impaired function, common in both conditions, favors its occurrence.^16^^,^ 17 ^,^ 29

Lam-Himlin et al.^9^ prospectively analyzed, within one-year interval, gastric biopsies performed by endoscopy and gastrectomy specimens with identification of *Sarcina ventriculi*. Besides, they retrospectively analyzed gastric biopsies and surgical specimens of patients with history of duodenal or pyloric mass or ulcer, in the previous twenty years. *Sarcina ventriculi* could be identified in six biopsies, from five different patients (two biopsies from the same patient), always associated with gastric emptying obstruction or delay, only in one case the bacterium was identified inside the mucosa associated with acute inflammation and ulcer. PCR and DNA sequencing were performed to confirm the bacterial presence.

Haroon Al Rasheed et al.^6^ reported a case with identification of the bacterium in a gastric ulcer after treatment and eradication of *Helicobacter pylori*. The two bacteria, however, can coexist, as described by Sauter et al.,^11^ who reported the cases of siblings presenting gastric symptoms. In both cases, upper gastrointestinal endoscopy demonstrated edematous pylorus, within other findings, and they had *Sarcina ventriculi* identified, as well as *H.* pylori. Also, its coexistence with *Candidas sp*. has been described in four patients, and the coexistence with *Giardia intestinalis* in another patient.^8^^,^ 15 ^,^ 17 ^,^ 32 ^,^ 33

There is no established standard treatment, and hemodynamic and respiratory supports must be initiated according to the patient clinical presentation. If there is no mucosal damage diagnosed at endoscopy, and the patient’s symptoms can be related to other etiology, the use of antibiotics is not mandatory.^23^ Fasting seems to contribute to clinical improvement by eliminating the source of carbohydrates to the bacteria, and the antibiotic therapy regimens are varied and may be used orally or intravenously, associated or not with proton-pump inhibitor and prokinetic.^15^^,^ 46 The most commonly used regimen is the combination of metronidazole and ciprofloxacin and it was prescribed in twelve cases (25%). Other schemes included metronidazole, clarithromycin, gentamicin, vancomycin, imipenem, amoxicillin, fluoroquinolone, fluconazole and amphotericin B.^10^^,^ 46 ^,^ 49

There is an increase in reports of the bacteria identification in the last years. This raises the question of whether its incidence is actually increasing, or if more attention to its diagnosis is being given recently. The retrospective analysis of gastric biopsies and specimens, performed by Lam-Himlin et al.,^9^ over twenty years, suggests a reappearance in humans, and this could be related to several factors, such as bacterial selection, changes in eating habits or food hygiene. Although there is not enough evidence to prove the pathogenicity of the bacteria on the alimentary tract mucosa, the reported cases presenting with emphysematous gastritis and gastric perforation suggest its contribution to aggravation in a pre-existing ulcer lesion. Furthermore, including the case in our hospital, there were four fatal outcomes associated with the bacterial identification (9%).^35^^,^ 40 ^,^ 43

Among the two cases described by our group, one presented a fatal outcome. Probably, if the patient was evaluated with upper gastrointestinal endoscopy prior to the circulatory shock, and the diagnosis of gastric ulcer had been performed earlier, the outcome would be different. We do not know the patient's detailed medical history, however, there was a report by a family member of sporadic use of omeprazole, suggesting previous dyspepsia. The second case follows the previously described relationship between mechanical obstructive factor, accumulation of residues and acidic environment rich in carbohydrates and low pH, contributing to the bacterial proliferation.

## CONCLUSION

In summary, the true pathogenicity and the real prevalence of the bacteria in humans are not completely understood. Despite this lack of knowledge, it may be related to serious complications, especially when associated with pre-existing mucosal damage. The increasing number of case reports may suggest a reappearance in humans. In conclusion, according to the few evidences we have, the presence of *Clostridium ventriculi* (formerly *Sarcina ventriculi*) should be considered as a marker for slowed food transit, and the etiology of this condition should be investigated.
